# Supplementary material for: Field Investigation of Effect of Plants on Cracks of Compacted Clay Covers at a Contaminated Site
Source: Int J Environ Res Public Health. 2022 Jun 13;19(12):7248. doi: 10.3390/ijerph19127248 (PMC9223466; doi:10.3390/ijerph19127248)
Supplement: Supplementary file 1 [file ijerph-19-07248-s001.zip › ijerph-1731989-supplementary.pdf]

Table S1. Main target contaminants in different depth of unsaturated zone

Test point	Target Pollutants	Contaminated Depth (m)	Contaminated Area (m^2)	Maximum concentration (mg/kg)
T-18, T-19, J-10	1-Naphthylamine	7	141	1180
	TPH	7		1470
J-13	1-Naphthylamine	8	161	13800
	Naphthalene	9		205
J-9, J-12	TPH	7	130	9880
	Chlorobenzene	6		618
J-15, J-16, J-11, T-22	Chlorobenzene	5	610	447
	1,2-Dichlorobenzene	5		675
	1,3-Dichlorobenzene	6		91.9
J-14, T-23, T-21	Dichlorobenzene	5	320	218
	Chlorotoluene	5		9.86
T-20	Naphthalene	5	62	270
	1-Naphthylamine	5		1630
T-24, T-15, J-8	TPH	5	71	14370
T-12, J-5	Naphthalene	10	83	265
	Chlorobenzene	9		106
T-4, J-2, T-7	TPH	8	316	1240
	1-Naphthylamine	7		990
J-3, J-6, T-5, T-8, T-11, T-9, J-7, J-4	Chlorobenzene	6	7315	8840
	1,2-Dichlorobenzene	5		460
	1,3-Dichlorobenzene	5		139
	1,4-Dichlorobenzene	6		296
	Chlorotoluene	7		29.02
	1-Naphthylamine	5		276
	TPH	5		9020
T-1, J-1, T-3, T-6, T-2	1,3-Dichlorobenzene	6	2000	10.4
	TPH	8		3240
